# Case Report: The aslanger pattern as an indicator of severe acute coronary syndrome

**DOI:** 10.3389/fcvm.2025.1633562

**Published:** 2025-12-08

**Authors:** Ying Zhu Chen, Zheng Yang Yao, Li Wen Lin, Qiao Yun Luo, Rong Li, Xin Jun Zhao

**Affiliations:** 1 Guangzhou University of Chinese Medicine, Guangzhou, China; 2 Department of Cardiovascular Medicine, The First Affiliated Hospital of Guangzhou University of Chinese Medicine, Guangzhou, China

**Keywords:** aslanger pattern, acute coronary syndrome, acute coronary occlusion, percutaneous coronary intervention, case report

## Abstract

Current guidelines categorize acute coronary syndrome (ACS) into unstable angina, ST-segment elevation myocardial infarction (STEMI), and non-ST-segment elevation myocardial infarction (NSTEMI). However, this categorization paradigm can lead to the underrecognition of acute coronary occlusion (ACO), especially when electrocardiographic findings are atypical. The Aslanger pattern is a critical ECG signature characterized by isolated ST-segment elevation in lead III with concomitant ST depression in leads V4–V6. It is often associated with severe multivessel disease, poor prognosis, and high mortality. We present two cases of chest pain with ECGs consistent with the Aslanger pattern. Coronary angiography confirmed three-vessel disease, with ACO in at least one vessel and severe stenosis in another. Notably, Patient 1 suffered in-hospital mortality, while Patient 2 had an uneventful recovery, underscoring the impact of timely intervention. This report emphasizes the value of recognizing the Aslanger pattern to bridge a diagnostic gap in ACS, enabling early identification of ACO, prompt reperfusion therapy, and ultimately, improved patient outcomes.

## 1 Introduction

Acute coronary syndromes (ACS) encompass unstable angina, ST-segment elevation myocardial infarction (STEMI), and non-ST-segment elevation myocardial infarction (NSTEMI) (1). Current guidelines define STEMI by specific electrocardiographic (ECG) criteria, including new ST-segment elevation in contiguous leads or a new-onset left bundle branch block (LBBB) (2). A significant limitation of this paradigm is that it fails to identify over a quarter of patients with acute coronary artery occlusion (ACO), potentially delaying life-saving reperfusion therapy (3). This underscores a significant limitation in the current ACS diagnostic paradigm. The Aslanger pattern is a specific ECG appearance first reported in patients with NSTEMI and is meant to represent severe occlusive coronary artery disease. Its characteristic features include: ST-segment elevation isolated to lead III (without elevation in other inferior leads); ST-segment depression in leads V4–V6 (without depression in lead V2); and a greater magnitude of ST-segment elevation in lead V1 than in lead V2 (4). We report two patients with ACS whose ECGs conformed to this pattern. Coronary angiography in both cases revealed severe multi-vessel disease with acute occlusion. The stark contrast in their outcomes—in-hospital mortality vs. uneventful recovery— highlights the urgent need for increased clinical vigilance regarding the Aslanger pattern. Its early identification is pivotal for prompting timely intervention, thereby avoiding diagnostic pitfalls and potentially improving patient survival.

## 2 Case description

### 2.1 Case 1

**Figure 1:**
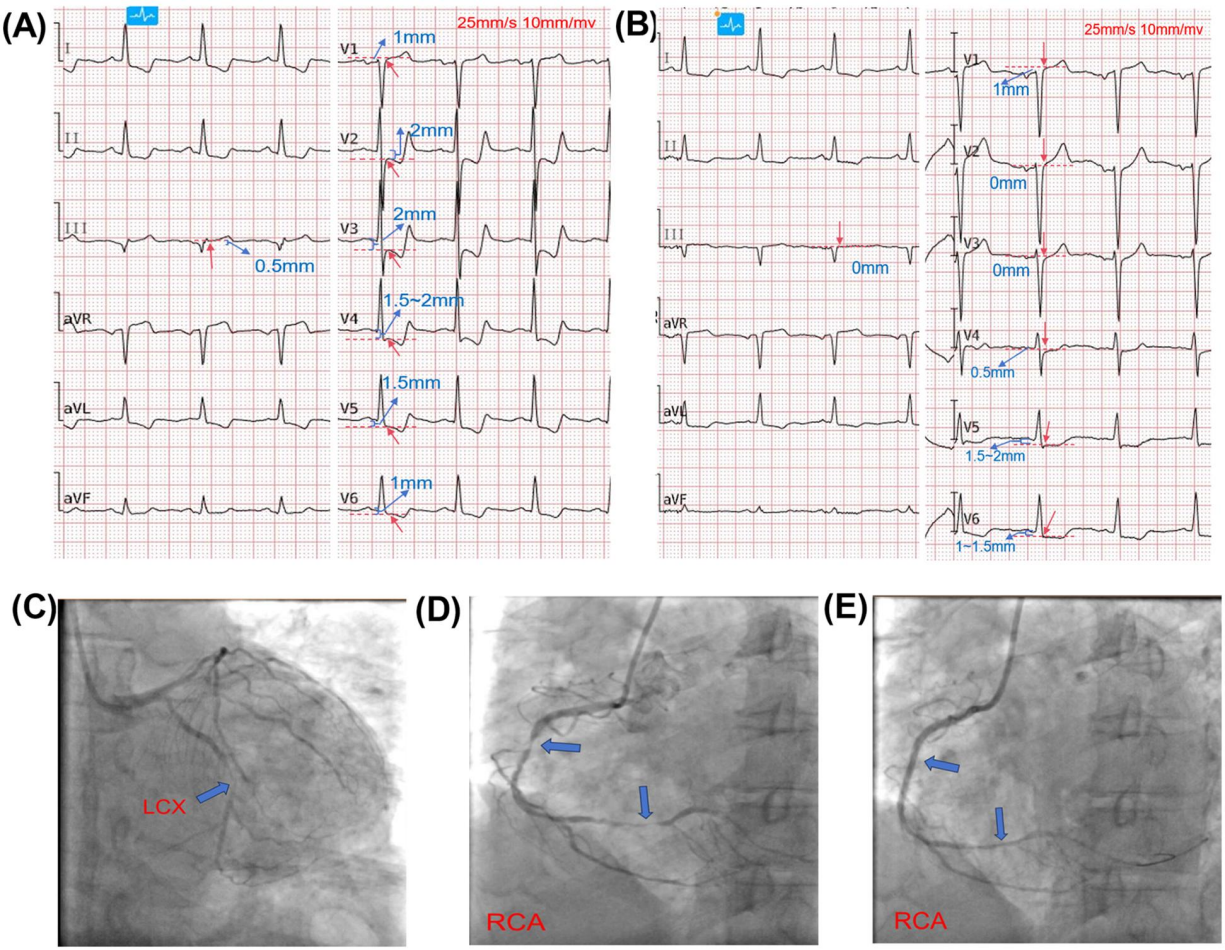
Electrocardiographic and angiographic findings. Red arrows indicate the J-point on ECG; red dashed lines and blue text denote ST-segment deviation. Red labels on angiograms indicate coronary arteries; blue arrows indicate lesion locations. **(A)**: Initial ECG showing ST-segment elevation in lead III, ST-segment depression in leads I and II, and ST-segment depression in leads V2–V6 with terminal T-wave positivity. **(B)**: Post-procedural ECG showing normalized ST segments in lead III and V2–V3, with persistent though improved ST-segment depression in V4–V6. **(C)**: Angiogram of LAD and LCX. The LCX showed diffuse plaques with 90%–95% stenosis in its mid-segment (length: 15 mm; RVD: 2.5 mm; TIMI flow grade III). The LAD had multiple plaques from proximal to mid-segment (RVD: 2.5 mm; lesion length: 20 mm). The first diagonal branch (D1) exhibited 90%–95% diffuse stenosis (RVD: 1.5 mm; length: 30 mm; TIMI III).**(D)**: RCA angiography revealed 60%–90% stenosis in the mid-segment (RVD: 2.5 mm) and 80%–95% diffuse stenosis just before the distal bifurcation, with extensive calcification (TIMI III).**(E)**: RCA after revascularization, showing restored flow.

A 56-year-old female patient presented with chest discomfort that worsened with activity and lasted for 6 h before she developed significant, persistent chest pain in the precordial region with no extension to other areas, accompanied by sweating, nausea, vomiting, and epigastric distension. Past medical history included hypertension, diabetes mellitus, and hyperthyroidism. The patient had no family history of heart disease. Physical examination revealed no significant abnormalities. An emergency electrocardiogram suggested ST-segment elevation in lead III, ST-segment depression in leads I and II, and ST-segment depression in leads V2-V6 with terminal T-wave positivity (Figure 1A). Laboratory tests showed myoglobin 122.5 ng/mL, ultrasensitive troponin I 0.249 ng/mL, brain natriuretic peptide (BNP) 181.7 pg/mL, and creatinine 214 μmol/L. NSTEMI was diagnosed. Percutaneous coronary intervention (PCI) was performed after administration of clopidogrel bisulfate and aspirin enteric-coated tablets loaded with antiplatelet aggregates. Coronary angiography revealed multiple plaques in the proximal to mid segment of the LAD, with a reference vessel diameter (RVD) of approximately 2.5 mm and a lesion length of 20 mm. The first diagonal branch (D1) exhibited 90%–95% diffuse stenosis, with an RVD of 1.5 mm and a lesion length of 30 mm (TIMI flow grade III). The LCX showed diffuse plaques with 90%–95% stenosis in its mid-segment, measuring 15 mm in length, and an RVD of 2.5 mm (TIMI flow grade III) (Figure 1C). The RCA demonstrated 60%–90% stenosis in its mid-segment (RVD 2.5 mm) and 80%–95% diffuse stenosis immediately preceding the distal bifurcation, accompanied by extensive calcification (TIMI flow grade III) (Figure 1D). A 2.5 × 24 mm rapamycin-eluting stent was implanted in the mid RCA, achieving final TIMI grade III flow(Figure 1E). The patient's postoperative electrocardiogram suggested normalized ST segments in lead III and V2–V3, with persistent though improved ST-segment depression in V4–V6 (Figure 1B). Echocardiography confirmed severely reduced left ventricular ejection fraction (LVEF of 26%), mitral valve prolapse with regurgitation, hypokinesis of the apical, anterior, and anteroseptal segments, and mildly reduced motility of the mid-lateral and inferior walls. Postoperative testing for troponin I was elevated to 72.67 ng/mL, BNP was 13,103.2 pg/mL, and serum creatinine was 798 μmol/L. By analyzing the etiology, the patient may have experienced complications of acute myocardial infarction, that is, the patient developed heart failure, malignant arrhythmia, ventricular aneurysm, and acute renal failure Postprocedurally. Based on the serum creatinine test results, the acute kidney injury has reached stage 3. The renal failure may have been due to contrast media. The treatment plan included antiplatelet aggregation, diuresis, improvement of heart failure, and continuous renal replacement therapy.

**Figure 2:**
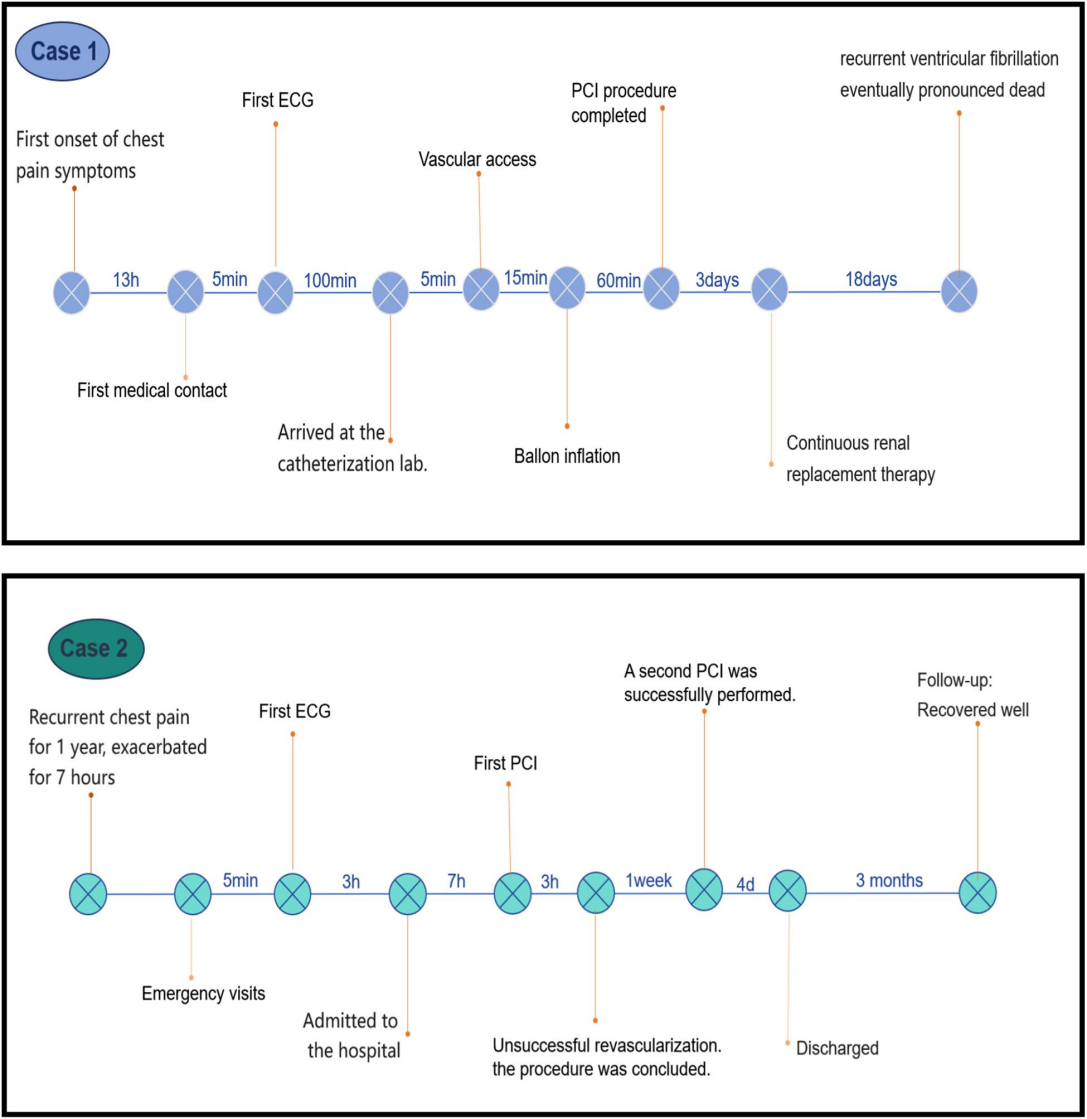
Timelines of key clinical events for case 1 and case 2.

Upon admission, the patient had a GRACE score of 186 and was in Killip class III. Subsequently, the patient developed sustained ventricular fibrillation, which may have been a peri-procedural complication or a consequence of acute kidney injury. The occlusion lesion in the right coronary artery, which affects the blood supply to the SA nodal artery and conus artery, increases the incidence of arrhythmic complications. Resuscitation measures included electrical cardioversion, antiarrhythmic pharmacotherapy (epinephrine, atropine, lidocaine, amiodarone), cardiopulmonary resuscitation, and mechanical ventilation. Due to financial constraints, mechanical circulatory support such as an intra-aortic balloon pump (IABP), Impella, or extracorporeal membrane oxygenation (ECMO) was not administered. Despite 90 min of sustained resuscitation efforts, sinus rhythm was not restored, and the patient succumbed to the condition (Figure 2).

### 2.2 Case 2

**Figure 3:**
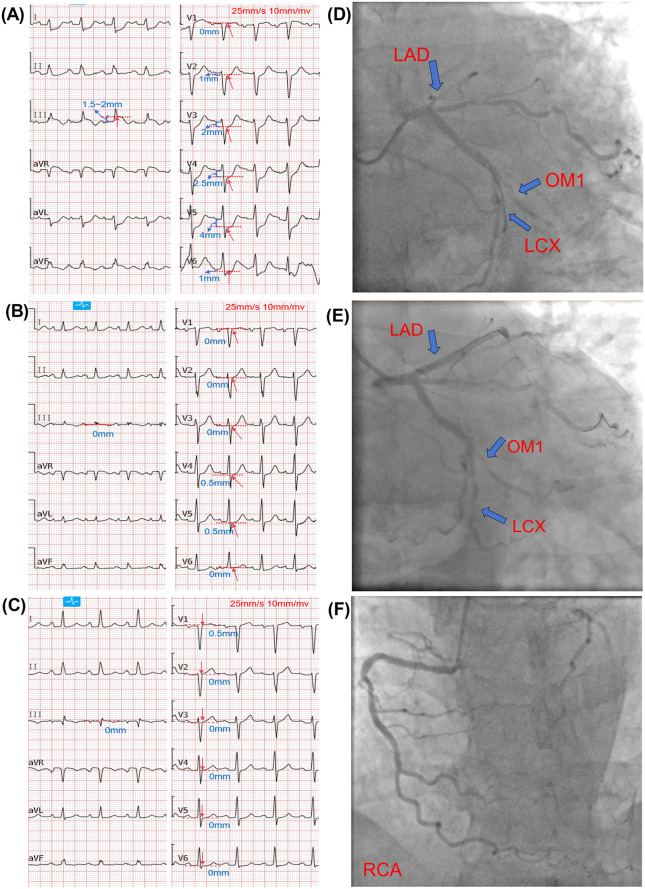
Electrocardiographic and angiographic findings (case 2). Red arrows indicate the J-point on ECG; red dashed lines and blue text denote ST-segment deviation. Red labels on angiograms indicate coronary arteries; blue arrows indicate lesion locations. **(A)**: Emergency department ECG showing ST-segment elevation in leads III, aVR, and V1; ST-segment depression with positive terminal T-waves in leads I, aVL, and V2–V6; and absence of ST-segment elevation in leads II and aVF. **(B)**: Pre-procedural ECG showing resolution of ST-segment deviations in most leads, with only mild residual ST depression in leads V4–V5. **(C)**: Post-procedural ECG showing mild residual ST elevation in lead V1, with complete normalization in all other leads. **(D)**: Pre-PCI angiogram showing 20%–30% stenosis at the left main (LM) ostium; 90%–95% diffuse stenosis in the proximal LAD (RVD 3.0 mm, lesion length 55 mm, heavy thrombus burden, TIMI grade II flow); total occlusion of the proximal LCX (TIMI grade 0); and 60%–70% stenosis in the proximal OM1. **(E)**: Post-PCI angiogram showing significantly improved stenosis in the LCx and OM1 (TIMI grade III flow) and resolution of stenosis in the proximal to mid LAD (TIMI grade III flow). **(F)**: Angiogram of the right coronary artery (RCA).

A 60-year-old male had a medical history of hypertension for > 1 year and a 10-year history of smoking. His chief complaint on admission was paroxysmal chest pain with cold sweats for 1 year, which was aggravated for 7 h. ECG examination in the emergency room showed ST-segment elevation in leads III, aVR, and V1; ST-segment depression and positive T-wave terminals in leads I, aVL, and V2–V6, and no ST-segment elevation in leads II and aVF (Figure 3A). Ultrasensitive troponin I was 0.052 ng/mL (The normal range is 0–0.034 ng/ml), and BNP precursor was 949.4 pg/mL. The patient was hospitalized for acute-on-chronic heart failure. However, the patient's ultrasensitive troponin I was elevated at 38.53 ng/mL after admission. An electrocardiogram performed at this time showed resolution of ST-segment deviations in most leads, with only mild residual ST depression in leads V4–V5 (Figure 3B). The patient had a GRACE score of 231 and was in Killip class IV. The patient was later diagnosed with acute myocardial infarction. PCI was performed after administration of loading doses of aspirin and clopidogrel. Angiography indicated 20%–30% stenosis at the left main (LM) ostium, 90%–95% diffuse stenosis in the proximal LAD (RVD 3.0 mm, lesion length 55 mm, heavy thrombus burden, TIMI grade II flow), total occlusion of the proximal LCX (TIMI grade 0), and 60%–70% stenosis in the proximal OM1 (Figure 3D). The RCA had 40%–50% stenosis (TIMI grade III) (Figure 3F). During the initial procedure, percutaneous transluminal coronary angioplasty (PTCA) was performed on the LAD and LCX using multiple balloons (1.5 × 10 mm, 2.0 × 15 mm, 2.5 × 15 mm).

Due to the heavy thrombus burden and poor distal flow (TIMI grade II in LAD and TIMI grade 0 in LCX), which substantially increased procedural risk, along with concerns that prolonged procedure time and contrast exposure could worsen the patient's already compromised renal function and clinical condition, particularly given concurrent acute heart failure with persistent acidosis, a staged revascularization strategy was adopted. One week later, IVUS-guided PCI of the LAD was performed, which revealed 90%–95% diffuse stenosis with circumferential calcium. After pre-dilation and cutting balloon angioplasty, three rapamycin-eluting stents (3.0 × 18 mm, 3.0 × 23 mm, and 3.5 × 18 mm) were implanted sequentially, followed by post-dilation. Final angiography showed TIMI grade III flow.(Figure 3E). A postoperative review of the ECG showed that mild residual ST elevation in lead V1, with complete normalization in all other leads (Figure 3C). Echocardiography after the first PCI showed: impaired left ventricular systolic function (LVEF 28%), left ventricular enlargement (LVEDD 61 mm), thinning and near-akinesis of the apical and mid-lateral segments as well as part of the posterior wall, and significantly hypokinetic motion in the remaining segments. This case was diagnosed as acutely decompensated heart failure. The patient was managed with guideline-directed medical therapy for heart failure in addition to secondary prevention medications. Subsequently, the patient had no further chest pain or serious complications and was discharged from the hospital after receiving a total of 11 days of secondary prevention of coronary artery disease and other treatments (Figure 2).

The patient was discharged on a comprehensive guideline-directed regimen for heart failure and secondary prevention, including antiplatelet therapy with aspirin (100 mg once daily) and clopidogrel (75 mg once daily), lipid-lowering therapy with atorvastatin (20 mg once daily) and ezetimibe (10 mg once daily), as well as heart failure-specific medications consisting of sacubitril/valsartan (50 mg twice daily), metoprolol succinate (71.25 mg once daily), spironolactone (20 mg once daily), and furosemide (20 mg twice daily). Additional medications included pantoprazole, magnesium and potassium aspartate, QiShenYiQi Dripping Pills (a traditional Chinese medicine), digoxin (0.125 mg once daily), liraglutide, and insulin aspart 30 mix for glycemic control. The patient was followed up at 30 days post-discharge and was recovering well, continuing the aforementioned medication regimen. A 3-month follow-up echocardiogram showed significant improvement in cardiac function, with the left ventricular ejection fraction recovering to 48%.

## 3 Discussion

The Aslanger pattern is a special type of ECG presentation of inferior wall myocardial infarction with extensive subendocardial ischemia, proposed by Emre Aslanger et al. in 2020; it has an incidence of approximately 6.3% in 1,000 patients with NSTEMI and is often accompanied by severe coronary artery lesions and high mortality (4). The Aslanger pattern is likely due to the presence of an inferior wall myocardial infarction combined with extensive subendocardial ischemia due to the stenosis of at least one non-infarcted coronary artery. This finding suggests the presence of multiple coronary artery disease. The associated infarcted arteries were more commonly the LCX and RCA, with a higher incidence in the LCX.

ST-segment elevation in specific leads typically indicates the ischemic region affected by the culprit lesion. However, extensive subendocardial ischemia does not occur in a single direction on the ECG vectors. The electrocardiographic manifestation of diffuse subendocardial ischemia consists of extensive and significant ST-segment depression (usually >0.5 mm or 1 mm), which may be accompanied by T-wave inversion, with possible mild ST-segment elevation in lead aVR. The underlying pathophysiology may include severe hypotension, significant anemia, or critical multivessel stenosis. Left main coronary artery (LMCA) occlusion represents the most severe form, characterized by more pronounced ST-segment depression (STD), particularly in leads V3–V5 (most prominent in V4), along with marked ST-segment elevation (STE) in leads V1 and aVR (5). When the ischemic area involves the inferior wall and subendocardium, as seen in the Aslanger pattern, the extensive subendocardial ischemia can mask the vector of inferior ischemia. This causes the combined electrical vector to shift rightward, perpendicular to lead aVF. Consequently, the Aslanger pattern displays ST-segment elevation only in lead III, with ST-segment depression in leads I and II (4, 6).

The ECGs of both patients were consistent with the Aslanger pattern, characterized by ST-segment elevation in lead III, ST-segment depression in leads I and II, a nearly isoelectric ST-segment in lead aVF, and ST-segment depression in leads 2 and V4–V6 with a positive T-wave endpoint (Table 1). In addition, there was a rapid increase in ultrasensitive troponin I levels within 24 h. Coronary angiography revealed severe stenosis or even occlusion in each case, and both patients experienced serious perioperative complications—decompensated heart failure and malignant arrhythmias. However, the ECGs of the above two cases were not identical to the standard Aslanger pattern, with the difference being that ST-segment depression was also present in leads V2–V3. This may be attributed to variations in the ischemic area and extent. When the ischemic extent is larger, the vectors are also shifted (7). Combined with the results of coronary angiography, this may be due to severe stenosis of the patient's LAD and its D1 branch, resulting in myocardial ischemia in the anterior wall. Thus, ST-segment depression in leads V2–V3 is also manifested on the ECG.

**Table 1:** ST-segment measurements at the J-point in each electrocardiographic lead (“+” indicates ST-segment elevation, “-” indicates ST-segment depression).

ECG leads	Case 1		Case 2		
Time point	Admission	Post-PCI	Admission	Pre-PCI	Post-PCI
V1	+1 mm	+1 mm	0 to +1 mm	0 mm	+0.5 mm
V2	−2 mm	0 mm	−1 to −1.5 mm	0 mm	0 mm
V3	−2 mm	0 mm	−2 mm	0 mm	0 mm
V4	−1.5 to −2 mm	−0.5 mm	−2.5 mm	−0.5 mm	0 mm
V5	−1.5 mm	−1.5 to −2 mm	−4 mm	−0.5 mm	0 mm
V6	−1 mm	−1 to −1.5 mm	−1 mm	0 mm	0 mm
I	−1 to−1.5 mm	−1 mm	−2 to −3 mm	0 mm	0 mm
II	−1 mm	−0.5 to −1 mm	0 to −0.5 mm	0 mm	0 mm
III	+0.5 to +1 mm	0 mm	+1.5 to +2 mm	0 mm	0 mm
aVR	+1 to +1.5 mm	+1 mm	0 to +1 mm	0 mm	0 mm
aVL	−1 mm	−0.5 to −1 mm	−2 to −3 mm	0 mm	0 mm
aVF	0 mm	0 mm	0 mm	0 mm	0 mm
QTc	438 ms	433 ms	473 ms	458 ms	438 ms
PR	161 ms	153 ms	168 ms	180 ms	216 ms
QRS	99 ms	106 ms	110 ms	97 ms	102 ms

The uniqueness of these two cases lies in the fact that, despite both exhibiting the Aslanger pattern, Patient 1 progressed rapidly to death even after timely intervention, highlighting the grave prognosis associated with Aslanger-positive ACS. In Case 2, the upsloping ST-segment depression in the precordial leads created a diagnostic challenge, as it bore resemblance to the De Winter pattern.The De Winter pattern is a distinct ECG entity characterized by J-point depression of 1–3 mm in precordial leads V1–V6, upsloping ST-segment depression, tall symmetrical T waves, and frequently poor R-wave progression. The culprit vessel is typically the left anterior descending artery (LAD). It is recognized as an STEMI-equivalent pattern mandating immediate coronary angiography and reperfusion therapy (8).

The key distinctions are as follows: The Aslanger pattern features ST-segment elevation in lead III. While the precordial ST segments may show upsloping depression, the T waves are different; they are not as symmetrically tall and peaked as in the De Winter pattern and may appear biphasic with a positive terminal phase. The culprit vessel in the Aslanger pattern is most often the left circumflex artery (LCX). Consequently, unless there is concomitant LAD disease, R-wave progression in the precordial leads usually remains normal (Table 2). Crucially, despite these differences, both patterns represent forms of ACS that require urgent percutaneous coronary intervention.

**Table 2:** Comparison between de winter pattern and aslanger pattern.

Feature	Aslanger pattern	de Winter pattern
ECG manifestations	ST-segment elevation only in lead III (no elevation in other inferior leads) ST-segment depression in V4–V6, but not in V2 ST-segment elevation in V1 > V2	1–3 mm upsloping ST-segment depression at the J point in leads V1–V6, continuing into tall, symmetrical T waves Often 1–2 mm ST-segment elevation in lead aVR QRS usually not or only slightly widened; poor R-wave progression may be present
Common culprit artery	Left Circumflex Artery (LCX) (more frequent)	Proximal Left Anterior Descending Artery (LAD)
Clinical implication	Suggests multivessel disease; early revascularization recommended	Considered STEMI-equivalent; requires immediate reperfusion

Guidelines (1) recommend emergency coronary angiography for patients with highly suspected ACS and unstable or persistent symptoms. However, the Aslanger pattern can be misdiagnosed in its early stages when troponin levels are not yet elevated or are only minimally elevated. It may be misinterpreted as nonspecific early ischemic changes or dismissed as indicative of non-urgent chronic coronary stenosis, leading to detrimental delays in reperfusion therapy. This is illustrated in a case report by Liu M-h et al., where a patient was initially diagnosed with intermediate-risk NSTEMI and underwent coronary angiography only two days after admission, which subsequently revealed critical stenosis in the left main (LM) and left anterior descending (LAD) arteries (7). Eiji et al. demonstrated that lesions tend to be more severe in most patients with the Aslanger pattern, as evidenced by the use of mechanical circulatory support and an elevated proportion of patients who die in the hospital (9). The Aslanger pattern should be considered to carry a risk equivalent to ST-elevation myocardial infarction (STEMI). Therefore, an early definitive diagnosis is crucial for the treatment and prognosis of ACS. Early revascularization is recommended to restore coronary blood flow, save the injured and dying myocardium, and reduce the size of the infarcted area.

Certainly, the Aslanger pattern possesses inherent limitations. As exemplified by Case 2, despite persistent chest pain and a progressive elevation in high-sensitivity troponin I levels following admission, a subsequent ECG revealed a partial resolution of ST-segment deviations in the affected leads. This ECG evolution generated a misleading impression of clinical improvement. Crucially, however, the decision for immediate reperfusion was guided by the integration of persistent symptoms and serial cardiac biomarker trends. This observation underscores that the pattern is not invariably static during ongoing ischemia or occlusion; it may fluctuate with dynamic ischemic changes. Moreover, the markedly disparate outcomes between the two presented cases illustrate that the precise relationship between this ECG signature and the temporal onset, evolutionary trajectory, and ultimate severity of ACS remains incompletely elucidated.

The STEMI/NSTEMI diagnostic paradigm has made the presence of ST-segment elevation almost synonymous with emergency reperfusion therapy for ACO (2). However, this association has never been formally investigated in randomized controlled trials. Aggregated accuracy data from one study reveal that classic STEMI criteria identified only 44% of angiographically confirmed ACOs. The same study demonstrated that incorporating a broader set of occlusion-specific indicators—such as hyperacute T waves, the de Winter pattern, the Aslanger pattern, and QRS distortion—increased the sensitivity for detecting ACO to 78%, with only a modest decrease in specificity to 94.4% (10). It is well-established that NSTEMI patients with an underlying ACO face a higher risk of mortality and major adverse cardiac events, necessitating early revascularization (3). Conversely, not all patients who meet STEMI criteria actually have an ACO (11). In such cases, the benefit of urgent reperfusion therapy remains debatable. It must be emphasized that the pathological condition requiring urgent reperfusion is the ACO itself, not the millimeter-based ECG criteria that may or may not be present. Consequently, a prevailing view is that the STEMI/NSTEMI classification of acute coronary syndromes has inherent diagnostic limitations, underscoring the urgent need for a more comprehensive model to accurately identify ACO (12).

Currently, the acute total coronary artery occlusion (OMI)/incomplete coronary artery occlusion (NOMI) pattern is receiving more attention (13). OMI was defined as an acute culprit lesion with TIMI 0–2 flow, or an acute culprit lesion with TIMI 3 flow intervened upon and with highly elevated troponin (cTnI > 10.0 ng/mL, hs-cTnI > 5,000 ng/L) (though the absence of an occlusive lesion on angiography does not exclude OMI, due to the possibility of spontaneous reperfusion prior to the procedure) (14). This framework explicitly establishes the gold standard as the angiographic finding, rather than reliance on minor deviations on the ECG. This represents a fundamental shift from pattern matching to pathophysiology, redirecting focus toward the actual pathological changes in the affected vessels and myocardium. The DIFOCCULT Study demonstrated that NSTEMI patients exhibiting the Aslanger pattern had a higher composite rate of ACO. The OMI/NOMI pattern was superior to the STEMI/NSTEMI pattern in terms of ECG diagnostic accuracy in predicting ACO and long-term mortality (15). Numerous scholars have proposed new insights or predictive methods to refine the OMI/NOMI model. For instance, an analysis of five clinical cases of atypical AMI with ACO integrated the OMI/NOMI framework with Bayesian reasoning, illustrating how ECG patterns like the Aslanger sign, hyperacute T waves, and the de Winter pattern can identify occult occlusions missed by traditional STEMI criteria (16). Nevertheless, owing to its complexity and the challenge of standardizing diverse atypical presentations, there are some limitations to the accurate utilization and generalizability of this pattern in clinical applications.

In conclusion, the Aslanger pattern improves the prediction of ACS, alerting physicians to the potential need for timely reperfusion therapy. The recognition of characteristic ECG findings such as the Aslanger pattern, hyperacute T waves, and the de Winter sign is crucial for the early diagnosis of ACS and for preventing diagnostic errors. Future large-scale studies to determine the diagnostic specificity and sensitivity of the Aslanger pattern could significantly complement current ACS guidelines. Ultimately, devising a singular, comprehensive diagnostic method for OMI remains challenging. Therefore, a multimodal approach integrating clinical history, biomarkers, coronary CT, and angiography is essential to accurately confirm the presence of an acute coronary occlusion.

## Data Availability

The original contributions presented in the study are included in the article/Supplementary Material, further inquiries can be directed to the corresponding authors.

## References

[1] Rao SV O’Donoghue ML Ruel M Rab T Tamis-Holland JE Alexander JH 2025 ACC/AHA/ACEP/NAEMSP/SCAI guideline for the management of patients with acute coronary syndromes: a report of the American College of Cardiology/American Heart Association joint committee on clinical practice guidelines. Circulation. (2025) 151:e771–862. 10.1161/CIR.0000000000001309 40014670

[2] Morrow DA. The fourth universal definition of myocardial infarction and the emerging importance of myocardial injury. Circulation. (2020) 141:172–5. 10.1161/CIRCULATIONAHA.119.044125 31958242

[3] Khan AR Golwala H Tripathi A Bin Abdulhak AA Bavishi C Riaz H Impact of total occlusion of culprit artery in acute non-ST elevation myocardial infarction: a systematic review and meta-analysis. Eur Heart J. (2017) 38:3082–9. 10.1093/eurheartj/ehx418 29020244

[4] Aslanger E Yıldırımtürk Ö Şimşek B Sungur A Türer Cabbar A Bozbeyoğlu E A new electrocardiographic pattern indicating inferior myocardial infarction. J Electrocardiol. (2020) 61:41–6. 10.1016/j.jelectrocard.2020.04.008 32526537

[5] Nikus K Pahlm O Wagner G Birnbaum Y Cinca J Clemmensen P Electrocardiographic classification of acute coronary syndromes: a review by a committee of the international society for holter and non-invasive electrocardiology. J Electrocardiol. (2010) 43:91–103. 10.1016/j.jelectrocard.2009.07.009 19913800

[6] Aslanger EK. Reply to “acute myocardial infarction with ST elevation isolated to lead III (and aVR)”. J Electrocardiol. (2025) 89:153889. 10.1016/j.jelectrocard.2025.153889 39904108

[7] Liu M Li H Li A Liu R Liu H Gao L A patient with acute myocardial infarction with electrocardiogram aslanger’s pattern. BMC Cardiovasc Disord. (2024) 24:3. 10.1186/s12872-023-03678-x 38166569 PMC10763094

[8] de Winter RJ Verouden NJW Wellens HJJ Wilde AAM. A new ECG sign of proximal LAD occlusion. N Engl J Med. (2008) 359:2071–3. 10.1056/NEJMc0804737 18987380

[9] Eiji M Kota K Ryo A Daisuke T Hideto C Naoya O Clinical features of the aslanger pattern to compensate for the limitation of ST-elevation myocardial infarction (STEMI) criteria. Cureus. (2023) 15:e33227. 10.7759/cureus.33227 36601361 PMC9805815

[10] de Alencar Neto JN Scheffer MK Correia BP Franchini KG Felicioni SP Nogueira De Marchi MF. Systematic review and meta-analysis of diagnostic test accuracy of ST-segment elevation for acute coronary occlusion. Int J Cardiol. (2024) 402:131889. 10.1016/j.ijcard.2024.131889 38382857

[11] Hillinger P Strebel I Abacherli R Twerenbold R Wildi K Bernhard D Prospective validation of current quantitative electrocardiographic criteria for ST-elevation myocardial infarction. Int J Cardiol. (2019) 292:1–12. 10.1016/j.ijcard.2019.04.041 31056411

[12] Aslanger EK Meyers HP Bracey A Smith SW. The STEMI/NonSTEMI dichotomy needs to be replaced by occlusion MI vs. Non-occlusion MI. Int J Cardiol. (2021) 330:15–15. 10.1016/j.ijcard.2021.02.015 33577907

[13] Sankardas MA Ramakumar V Farooqui FA. Of occlusions, inclusions, and exclusions: time to reclassify infarctions? Circulation. (2021) 144:333–5. 10.1161/CIRCULATIONAHA.121.055827 34339305

[14] Kola M Shuka N Meyers HP Zaimi E Smith SW. OMI/NOMI: time for a new classification of acute myocardial infarction. J Clin Med. (2024) 13:5201. 10.3390/jcm13175201 39274412 PMC11395726

[15] Aslanger EK Yıldırımtürk Ö Şimşek B Bozbeyoğlu E Şimşek MA Yücel Karabay C DIagnostic accuracy oF electrocardiogram for acute coronary occlusion resulting in myocardial infarction (DIFOCCULT study). IJC Heart & Vasculature. (2020) 30:100603. 10.1016/j.ijcha.2020.100603 32775606 PMC7399112

[16] de Alencar JN Helseth H de Assis HM Smith SW. Bayesian Diagnosis of occlusion myocardial infarction: a case-based clinical analysis. Diagnostics. (2025) 15:2148. 10.3390/diagnostics15172148 40941636 PMC12427917

